# School Climate, Academic Achievement and Educational Aspirations in Roma Minority and Bulgarian Majority Adolescents

**DOI:** 10.1007/s10566-018-9451-4

**Published:** 2018-04-05

**Authors:** Radosveta Dimitrova, Laura Ferrer-Wreder, Johan Ahlen

**Affiliations:** 1 Department of Psychology, Stockholm University, Frescati Hagv. 14, 106 91 Stockholm, Sweden; 2 Department of Psychology, Uppsala University, Box 1225, 751 42 Uppsala, Sweden

**Keywords:** School climate, Academic achievement, Educational aspirations, Roma adolescents, Bulgaria

## Abstract

**Background:**

School climate can promote students’ academic achievement and high educational aspirations. School climate refers to the quality and character of school life, norms, values, social interactions and organizational processes within a school.

**Objective:**

We examined for the present sample whether (a) school climate relates to academic achievement and educational aspirations and (b) such relations vary for Roma minority compared to their majority peers.

**Method:**

Participants in this cross-sectional study were 356 adolescents aged 11–19 years old (159 Roma, 197 Bulgarian majority), 332 mothers (149 Roma, 183 majority), 231 fathers (104 Roma, 127 majority) and 221 majority teachers who completed self-report surveys to address the study goals. Adolescents provided data on educational aspirations and academic achievement, parents on their children’s educational aspirations and teachers reported on school climate. We employed linear mixed models to explore associations of school climate, academic achievement and educational aspirations among Roma and Bulgarian majority youth.

**Results:**

There were negative associations between teacher-reported school climate and students’ academic achievement, as well as adolescent and parental educational aspirations for Roma adolescents only. Roma adolescents and parents reported lower academic achievement and educational aspirations than their majority counterparts.

**Conclusions:**

This study supports the relevance of school climate in relation to academic achievement and aspirations of disadvantaged minority students. Interventions should pay close attention to perceptions and attitudes in a school to successfully promote positive outcomes among students.

## Introduction

Over the past three decades, school climate has been increasingly recognized as an important asset for promoting safer, supportive, and civil schools (Brand et al. 2003). Across nations, there is a growing global interest in school climate and an appreciation that when it is carefully measured and considered, the climate of schools can inform strategies to improve school management and students’ academic achievement (Espelage et al. 2014; Thapa et al. 2013). Although there is no consensus on a particular set of defining characteristics, school climate often refers to the quality and character of school life as it relates to norms and values, social interactions and organizational processes (Freiberg 1999), as well as the atmosphere of a school, the nature and quality of interpersonal relationships and communication patterns within the school (Welsh 2000). School climate has been consistently shown to play a major role in shaping students’ educational experience, and research has emphasized the importance of developing a positive school climate in order to promote school attendance, academic achievement, engagement and high educational aspirations (Bear et al. 2011; Brand et al. 2003).

In this study, we examined teachers’ perceptions of school climate and how such perceptions may have bearing on adolescents’ academic achievement and educational aspirations. Our participant groups were youth of Roma origin living in Bulgaria. Bulgaria is a post-communist country in Eastern Europe where many Roma suffer poor academic achievement, high school dropout, along with a lack of access to adequate social and educational services (Amnesty International 2007). It is common that Roma youth in Bulgaria will end their education at the end of primary school (i.e., 89%) and a minority (i.e., 10%) will end their education after secondary school (Ringold et al. 2005). Therefore, promoting resources for improving school climate and educational prospects for such a disadvantaged group is pressing.

This study extends prior research conducted in the United States, as well as a handful of available empirical studies on school climate conducted in non-English speaking countries and among ethnic minority communities in Europe. We add important evidence that can support the wider public interest in school climate among one of Europe’s largest and most vulnerable and marginalized minority by examining how school climate characteristics relate to educational achievement and future aspirations of Roma youth and their parents. In so doing, we use information from multiple informants (e.g., student, teacher and parents) to advance what is known about how school climate may be relevant to positive adolescent development.

### School Climate, Academic Achievement and Educational Aspirations

Extant research has consistently documented that school climate is linked to multiple outcomes for students, teachers, and schools (Bear et al. 2014). Positive school climate relates to a variety of assets such as academic achievement (Bryk et al. 2010) and academic success (Bear et al. 2011; Brand et al. 2003). In addition, research has shown that factors within the family can be important to adolescents’ well-being and positive academic performance. Parental aspirations for children’s educational attainment have been documented as a particularly important factor predicting children’s academic achievement (Spera et al. 2009). These aspirations act as standards for performance that organize and direct parents’ behaviors toward their children (Wentzel 1998). In contrast to parental educational expectations (i.e., what parents realistically expect in terms of how far their child will go with her or his education), aspirations refer to what parents hope for regarding educational prospects and careers of their children. Parental aspirations for children’s educational attainment are also significantly and positively related to children’s setting of academic goals, persistence in school, and college attendance (Bask et al. 2014).

Several theoretical frameworks have attempted to explain the complex relations between parental educational aspirations and children’s academic achievement. Social-psychological perspectives typically view educational aspirations as a product of beliefs and feelings about the world and one’s self as well as cultural, formal, and informal educational environments, and, interactions with significant others. For example, according to social cognitive theory (Bandura 1977, 1986), educational aspirations reflect one’s motivation to achieve or succeed, which is formed by a lifetime of observations and experiences. Like social cognitive theories, cultural-ecological theories view educational aspirations as socially and contextually developed dispositions or orientations (Bronfenbrenner 1977). Relatedly, structural or blocked opportunity perspectives view educational aspirations as dispositions that develop in response to the presence or lack of structural and institutional forces, including institutional inequalities (Corcoran 1995).

In a similar vein, the integrative model by Garcia Coll et al. (1996) posits that markers of social position often predict available opportunities for children and youths and elicits certain behaviors, which in turn affect developmental outcomes. The model suggests that ethnic minority students often find themselves in social positions where educational and career opportunities are inadequate or limited and combined with other challenges (e.g., poverty, family needs, and neighborhood pressures) which ultimately discourage them from pursuing goals that are valued in the mainstream culture.

Although the relation between parents’ aspirations and children’s academic achievement has been well documented, little research has assessed contextual factors such as school climate that might be important to parents’ aspirations for their children’s educational attainment. There was only one study we are aware of that examined parents’ educational aspirations for their children as a function of ethnicity, children’s academic performance, and parents’ perceptions of school climate. This cross-sectional study was conducted within an ethnically diverse sample of families from the United States and showed that all parents had relatively high educational aspirations for their children, and, that children’s academic performance and parental perceptions of school climate were significantly and positively related to parental aspirations (Spera et al. 2009). Of interest for the current study is that although the relation between parental aspirations and student achievement has been well documented, researchers have not assessed school climate factors that might be important to these relations and children’s educational achievement in an ethnically diverse European sample. This is critically important, because a growing body of research has indicated that positive school climate can be critical to health promotion efforts in schools (Cohen 2001).

This study provides a novel contribution to the research literature in furthering our understanding of Roma youth in a European context. In so doing, it addresses a pressing research topic for this group (i.e., school processes, educational aspirations and achievement). We know little if anything about Roma youth compared to other ethnic minorities and the sparsely available research has rarely investigated aspects of Roma students’ schools and their school experience, such as school climate, parents’ educational aspirations and young people’s academic achievement. We also add important findings to what is known about the joint role mothers’ and fathers’ educational aspirations as well as teachers’ perceived school climate may play in adolescents’ educational aspirations and achievement. In adding unique data from multiple informants to this scarcely researched area for Roma families, we also have the strength of having a comparison group of families who are in the Bulgarian mainstream society. Therefore, we attempted to document the ways in which school climate and parental educational aspirations may function as a resource for educational aspirations and achievement of Roma adolescents.

### Aims and Hypotheses

Although many of the issues addressed above are relatively well documented in samples from the United States, studies have rarely examined the associations of school climate, academic achievement and educational aspirations in a single study outside of the United States. We could not find any study, which has investigated these relations in Roma ethnic minority compared to majority youth in Europe. Therefore, we examined how young people’s academic achievement and educational aspirations may relate to teachers’ perceptions of school climate, as well as parental educational aspirations. We operationalized overall school climate as an aggregate from a teacher-reported survey that measures teachers’ perceptions of their school environment, which is in accordance with extant literature (Shinn 1990). Positive school climate has been shown to relate to school satisfaction and behavior (Elsaesser et al. 2013; Koth et al. 2008; Wang et al. 2010), but so far, we know little about how teachers in an Eastern European context perceive their school climate and how it relates to students’ educational aspirations and overall academic achievement in diverse ethnic groups.

Our aim was to expand on prior work documenting the potential importance of school climate for youth by proposing that how teachers perceive the climate of their school would relate to academic achievement and aspirations of their students, as well as parents’ educational aspirations for their children. A positive school climate should be important to higher achievement at school, and higher future educational aspirations. Further, in building upon the school climate literature, we also examined whether teacher perceptions of school climate are differentially related to Roma minority relative to majority students’ academic achievement and future educational aspirations.

Therefore, the first study hypothesis was that overall school climate would be significantly and positively associated with better academic achievement and aspirations (Hypothesis 1a). However, we expected that the pattern of these relations would vary by ethnic background. Given the generally strong discrimination, social exclusion and school dropout in Roma ethnic minority groups (World Bank 2014), we expected a weaker relation between school climate and academic achievement and educational aspirations for Roma youths (Hypothesis 1b). Furthermore, in light of highly disadvantaged academic context of Roma students (Ringold et al. 2005), we expected significant ethnic group differences, such as, on average, lower ratings of academic achievement and educational aspirations in the Roma minority group compared to the majority group (Hypothesis 2).

## Method

### Participants and Procedure

The present study draws on student, parent, and teacher self-reported survey data collected from five high schools during the 2013–2014 academic years in large urban districts in the Western and Southern Bulgaria. Bulgaria is a post-communist country situated in Eastern Europe and borders Romania, Greece, Macedonia, Serbia, Turkey and the Black Sea. Roma are the major ethnic minority group accounting for nearly one million people out of a national population of seven million (National Statistics Institute 2011). Roma are largely segregated within Bulgaria and experience ongoing discrimination and non-inclusive schools that systematically deprive Roma students of educational prospects. Roma students are also most likely to drop out before the end of basic schooling, in part due to the prevalence of racism in many schools and the inability of schools to meet their needs (UNICEF 2011).

The selected schools were representative of geographically and ethnically mixed populations across the country and were sampled as part of a larger ongoing study in these regions. Study participants were in total 1142 of whom 356 adolescents aged 11–19 years old (159 Roma minority and 197 Bulgarian majority), 332 mothers (149 Roma and 183 Bulgarian), 231 fathers (104 Roma and 127 Bulgarian). A total of 221 teachers with majority Bulgarian ethnic background at the selected schools were also included (see Table 1). Participants were recruited via the schools. All contacted schools agreed to participate and the teachers’ response rate was very high (up to 95%). The response rate among mothers (80%), fathers (70%), and youth (98%) was also high.

**Table 1:** Sample characteristics for Roma and majority adolescents and parents

	Roma minority			Bulgarian majority		
	Youth	Mothers	Fathers	Youth	Mothers	Fathers
	*n* = 159	*n* = 149	*n* = 104	*n* = 197	*n* = 183	*n* = 127
Age						
Mean (*SD*)	14.99 (1.83)	36.31 (5.16)	39.62 (6.12)	15.28 (2.02)	40.83 (7.28)	44.48 (6.23)
Gender (%)						
Female	48	–	–	49	–	–
Male	52	–	–	51	–	–
Family SES (%)						
Low	97			14		
Middle	3			53		
High	–			33		
Educational aspirations *M* (SD)	3.51 (.98)	3.74 (1.00)	4.06 (.89)	4.30 (.88)	5.55 (.68)	4.42 (.80)
	α = .88	α = .92	α = .92	α = .91	α = .92	α = .94
Academic achievement *M* (SD)	3.36 (.88)			4.56 (.98)		
	α = .89			α = .91		

Data on family socioeconomic status (SES) was reported by participating youth, mothers, and fathers. SES was computed by creating a composite score of the education level of both parents (primary, secondary, and university degree) coded in three levels of low, middle, and high (Oakes and Ross 2003). The adolescent groups in this study differed in terms SES with Roma youth having a lower SES than their majority counterparts, χ^2^ (2, *N* = 326) = 220.96, *p* < .001. The adolescent participant group did not differ in terms of age or gender.

Prior to data collection, local school authorities were informed about the purpose and method of the study to acquire their consent and participation. The study was part of a larger project on Roma ethnic minority groups in Bulgaria and was approved by the Bulgarian National Agency for Child Protection and the Swedish Regional Ethics Committee. Materials were distributed to all parents containing information about the study and parental consent forms. Active consent was used and upon receiving the signed parental consent forms, youth filled in the survey in their classroom during the school day. Parents filled in the questionnaires in their homes, whereas teachers filled in the school climate measure in the school. On average, the questionnaires took 30–45 min to complete.

### Measures

All measures were translated from English into Bulgarian by four bilingual speakers while adhering to the standard guidelines to ensure linguistic equivalence by applying culturally sensitive translation of items in a committee approach and assure applicability of item contents in the local culture (van de Vijver and Leung 1997). Two separate focus groups of teachers and students were involved in assessing the appropriateness of the translated measures. The questionnaires were presented only in Bulgarian, because Roma pupils typically acquire literacy skills exclusively in Bulgarian. All measures showed very good internal consistency across cultural groups and respondents (see Table 1).

#### Educational Aspirations

Were measured with the Future Goals and Aspirations Scale from the Student Engagement Instrument (SEI; Appleton et al. 2006). The scale measures future educational aspirations defined as cognitive subtype of school engagement (e.g., relevance of school to future aspirations, learning goals, educational goal orientation, investment in learning, value of learning). The scale consists of five items rated on a 5-point scale (1 = *strongly disagree* to 5 = *strongly agree*) with higher scores indicating higher educational aspirations. Sample items include “I plan to continue my education following gymnasium”, “School is important for achieving my future goals”. The same items with slight change in wording were administered to adolescents, mothers, and fathers. For the purposes of this study, we created average composite score of both parental reports on educational aspirations for their children as also suggested by a high positive correlation between mother and father scores, *r*(210) = .64, *p* < .001.

#### Academic Achievement

Adolescents provided information on their grade point average scores (GPA) on major academic subjects taught in local schools (math, Bulgarian language and reading, physics and biology). The range of two to six is used for evaluation in local schools, where two represents insufficient and six represents an excellent score. An average score of all school subjects was created as in indicator of overall academic achievement with higher scores indicating better achievement (see Table 1). We chose self-reported academic achievement based on prior research on the accuracy of students’ self-reported grades compared to their actual grades indicating a high correlation between the two (Kuncel et al. 2005). Reliability of self-reported grades as a good estimate of actual grades has also been reported in prior work with adolescent samples in Bulgaria (Bankov et al. 2006).

#### School Climate

School climate was measured by the Pedagogical and Social Climate in School (PESOC) questionnaire—Teacher Report. PESOC is used to evaluate the pedagogical and social climate of a school (Grosin 2004) targeting both cultural (e.g., objectives, values and expectations) and structural (e.g., content and forms of pedagogical activities) aspects of school life. The teacher-rated PESOC is composed of 67 items rated on a 4-point Likert scale (1 = *completely agree* to 4 =* completely disagree*) with higher scores indicating better school climate. Sample items were “Our principal has high demands and expectations for pupil academic results”, “There is a high degree of unity among teachers regarding the schools goals”, “Parents are always informed if a child behaves badly in school”, “I have the support of my colleagues in selecting teaching content and methods”, “In this school you feel that you can develop as an educator.” The validity and reliability of the PESOC have been explored in large teacher samples covering several regions in Sweden (Carlson 2004; Hultin et al. 2016) supporting a very good validity and reliability as an uni-dimensional measure of school climate. Good factorial validity and reliability of PESOC as an unidimensional measure of school climate was also documented in a Bulgarian teacher sample with alpha of .97 (Dimitrova et al. 2015).

### Data Analysis

Given the dependent structure of the data (i.e., youths were nested within schools), we used linear mixed models (LMMs; Verbeke and Molenberghs 2000) to test our hypotheses. LMMs handles the data dependent structure by including random effects, which model the variability among clusters, and thus, the LMM deals with the potential similarity among youth within clusters. We performed the LMMs as two-level models with youth nested within schools. The variables GPA, youth-rated educational aspirations, and parents’ reports on their children’s educational aspirations, respectively, served as dependent variables in separate LMMs. In all LMMs, school climate was specified as a school-level predictor. Ethnicity and SES were specified as individual-level predictors.

In a first step, to answer Hypotheses 1a and 2, we examined the main effects of school-climate, ethnicity, and SES in a series of LMMs only including main effects. In a second step, to answer Hypothese 1b, and to model differential associations between predictors and outcomes for Roma and majority youth, we added and evaluated the school climate × ethnicity and SES × ethnicity interaction effect. The school-climate variable, and the SES variable were standardized (*z*-transformed), to make meaningful interpretations of the main effects in the LMMs including interaction effects. To determine *p* values of the Beta-coefficients in the LMMs, we used the Satterthwaite approximation, which evaluates the effective degrees of freedom when only estimates of the variance are known (Satterthwaite 1946). The LMMs were performed in the R software program (R Core Team 2015) using the “lmerTest” package (Kuznetsova et al. 2015).

## Results

Preliminary analyses tested for cross-cultural equivalence on the measures of academic achievement and educational aspirations across Roma and majority groups. Structural equivalence was evaluated with Tucker’s phi coefficient (above .90 acceptable and above .95 excellent; van de Vijver and Leung 1997) and checked by comparing each group factor solution. The values of Tucker’s phi across groups ranged from .99 to 1.00 for measures of academic achievement and educational aspirations. We concluded that these measures showed very good structural equivalence across ethnic groups.

### School Climate

Table 2 presents details from the LMMs performed in this study. We found no significant main effect of school climate in any of the LMMs evaluating GPA, youth and parent-rated educational aspirations. Consequently, we did not find support for the expected association between school-climate and academic achievement and aspirations as specified in Hypothesis 1a. However, when including interaction effects in the LMMs evaluating GPA and youth-rated educational aspirations, we found significant school climate × ethnicity interaction effects. GPA and youth-rated educational aspirations were negatively associated with teacher-rated school-climate for Roma youth only. This result is consistent with Hypothesis 1b. No school climate × ethnicity interaction effect was found regarding parents’ reports on youth’s educational aspirations.

**Table 2:** Results from the linear mixed models (LMMs)

Outcome	Predictor	Step 1 LMM (only main effects)				Step 2 LMM (including interaction effects)			
		Coefficient (*B*)	*df* ^a^	*t* value	*p* value	Coefficient (*B*)	*df* ^a^	*t* value	*p* value
GPA	School climate	− .18	6.57	− 2.11	.075	− .05	3.06	− .53	.631
	Ethnicity (Roman)	− 1.08*	2.87	− 6.14	.010	− 1.03**	2.81	− 8.56	.004
	SES	.29***	19.61	5.35	< .001	.46***	10.51	5.63	< .001
	School climate × ethnicity	–				− .21*	135.30	− 2.03	.044
	SES × ethnicity	–				− .31**	173.85	− 2.92	.004
Y-EA	School climate	− .03	3.93	− .38	.756	.03	5.90	.43	.683
	Ethnicity (Roman)	− .50	2.24	− 2.89	.089	− .61*	2.96	− 4.69	.019
	SES	.16	2.27	1.93	.178	.24**	21.65	2.85	.009
	School climate × ethnicity	–				− .35**	9.92	− 3.21	.009
	SES × ethnicity	–				− .04	48.72	− .34	.734
P-EA	School climate	.03	3.92	.47	.661	.09	4.72	1.22	.282
	Ethnicity (Roman)	− .44	1.58	− 3.52	.100	− .43	1.50	− 3.72	.099
	SES	.25	1.75	2.49	.148	.28*	4.81	3.87	.013
	School climate × ethnicity	–				− .11	76.50	− 1.24	.220
	SES × ethnicity	–				.01	1.36	.07	.955

^a^Effective degrees of freedom (*df*) estimated using the Satterthwaite approximations

*GPA* grade point average, *Y-EA* youth-reported educational aspirations, *P-EA* parent-reported educational aspirations, *LMM* linear mixed model

*p < .05; **p < .01; ***p < .001

### Ethnic Group Differences in Academic Achievement and Educational Aspirations

As can be seen in Table 2, we found a significant negative main effect of ethnicity regarding GPA. This result indicated that Roma had lower grades than the majority youth as predicted by Hypothesis 2. In the first step of the LMMs, no significant main effect of ethnicity was found regarding educational aspirations. In the second step of LMMs (including interaction effects), we found a significant main effect of ethnicity for youth-rated educational aspirations. However, the interpretation of main effects in regression analyses including interaction effects with continuous variables are restricted. Specifically, this result indicated that typical Roma youth (i.e., with mean levels of SES and school climate) reported lower educational aspirations than typical majority (Bulgarian) youth.

### Socioeconomic Status

According to Table 2, we also found a significant positive main effect of SES regarding GPA, suggesting that overall, higher SES was associated with higher grades. No significant main effect of SES was found regarding educational aspirations. However, when including interaction effects, we found a significant positive main effect of SES for GPA (now interpreted as the effect for the majority youth), and a significant negative SES × ethnicity effect, indicating that Roma compared to the majority youth, showed weaker association between SES and GPA. We also found a significant positive main effect of SES regarding youth-rated and parent-rated educational aspirations (now interpreted as the effect for the majority youth). However, in these latter LMMs, there were no significant negative SES × ethnicity effects.

To achieve more clarity in these latter results, we conducted the same analyses (i.e., the LMMs including interaction effects), but this time with Roma ethnicity as the reference group. In these analyses, we found no SES main effect (i.e., interpreted as the effect for the Roman youth only) regarding GPA, *B *= .14, *t*(16.46) = 1.92 *p *= .073, youth-rated educational aspirations, *B *= .20, *t*(6.48) = 2.35 *p *= .054, or parent-rated educational aspirations, *B *= .29, *t*(1.60) = 2.23 *p *= .186. In summary, these results showed that SES was only associated with academic achievement and educational aspirations in the majority (Bulgarian) youth.

## Discussion

The current study aimed at examining the association between teacher-rated school climate and three important educational features (1) GPA, (2) youth-rated educational aspirations, and (3) parental reports of youth’s educational aspirations. Contrary to expectations, we did not find an overall positive association between teacher-rated school climate and our examined educational features. However, in line with our hypotheses, we found differential associations between school-climate and students’ GPA and aspirations. Specifically, we found a negative association among these variables for the Roma minority youth (whereas no significant association was found for the majority youth). This result was unexpected, given the widely established beneficial association between school climate and indicators of adjustment and academic engagement in youth (Bear et al. 2011; Bryk et al. 2010). The result is not easily explained, however, a few possible explanations are discussed below.

First, the negative associations found in the study raise the question whether our measure of teacher-rated school climate actually relates to the students’ experience of school-climate. One speculation is that teachers’ positive views of school climate may also reflect a neglect of the challenges schools face with different ethnic groups. Second, although the school climate measure adopted in this trial (i.e., the PESOC) has shown adequate factorial validity and reliability in a Bulgarian sample (Dimitrova et al. 2015), the predictive validity of PESOC has not yet been established in this specific cultural context. It is possible that PESOC does not capture all relevant aspects of school climate in Bulgarian schools with substantial ethnic diversity. Finally, school climate was entered as a school-level predictor meaning that all youths at a particular school obtained the same value. Even though we specified schools as random effects in our models (and thereby controlled for other “unknown” common school factors), it is possible that such average school-climate values hide important variation within schools.

The study also showed that young people belonging to a severely marginalized ethnic minority in Europe (World Bank 2014) had lower academic achievement and aspirations than the majority youth attending the same schools. This is consistent with previous research pointing towards poorer educational prospects for Roma in the Bulgarian society, relative to majority youth (Ringold et al. 2005). According to social-psychological models (Bandura 1977, 1986), educational aspirations of Roma students and parents may reflect their motivation to achieve and succeed in the context of marginalization and segregation experiences. Also in line with the cultural-ecological (Bronfenbrenner 1977) and structural or blocked opportunity perspectives (Corcoran 1995), educational aspirations for Roma youth are possibly due to structural and institutional inequalities as well as impoverished, isolated, and segregated environment that jointly discourage Roma from pursuing high educational aspirations and goals (Garcia Coll et al. 1996). Thus, the lower academic achievement and future aspirations of Roma are not surprising, given their specific context of this often severely marginalized ethnic group. Efforts to prevent the onset of academic difficulties and promote better education for Roma students need to be cognizant of conditions in the school environment that may be limiting for these students.

Finally, we also found interesting results regarding SES, which was differently associated with achievement and aspirations for Roma compared to the majority youth. Specifically, our results imply that SES was not a significant protective factor for academic achievement and aspirations in Roma, as it was for the majority youth in our study, and compared to what is typically found in other studies (e.g., Van Ewijk and Sleegers 2010). However, our result is consistent with evidence from a meta-analysis (Sirin 2005), where a low association between SES and academic achievement was found for minority compared to White students in the United States. Possibly, the reality of being a victim of discrimination and social exclusion may reduce the protective effect of SES on academic achievement and future educational aspirations. While this study is specific to Bulgaria, it has the potential to add more nuance to our understanding of the relation between school climate and the educational experiences of disadvantaged minority students.

## Limitations and Conclusions

The current investigation adds to prior research on school climate factors and its relations with educational achievement and aspirations by comparing Roma minority and majority youth in Bulgaria. Yet, the interpretation of these findings should only be applied to the sample under investigation, and await replication in other samples with further cross-sectional and longitudinal study designs. It is unclear whether and to what extent these patterns can be generalized to other Roma populations, or even youth of other ethnic origins. Another limitation is that multi-method studies of school climate (e.g., parent and student ratings) are important to complement these findings, which in this study was based on teacher-reports only. We suggest that future studies examine school climate from multiple perspectives including case studies to better integrate relevant school processes for students (Bocchi et al. 2014).

Finally, we did not measure perceived discrimination experiences of Roma students at participating schools. Although it is widely acknowledged that discrimination is embedded in the history of Roma groups, we could not assess the individually experienced discrimination of youth and parents in our sample. Relatedly, our cross-sectional study design limits our ability to establish the nature of associations, which could well be bidirectional, or due to unaccounted explanatory variables.

Despite these limitations, this is the first comparative study offering unique insights into school climate factors among ethnic minority and majority youth in Bulgaria, and how these factors relate to academic achievement and aspirations, as measured from the perspectives of adolescents and parents. Based on the current findings, it remains extremely important to pay close attention to perceptions and attitudes in a school setting that are relevant for ethnic minority populations. We found that teacher reported school climate was negatively associated with academic achievement and aspirations for Roma students only, in contrast to the more typical finding that school climate is positively associated with student academic success and socio-emotional developmental outcomes (Cohen et al. 2009). This study supports the relevance and salience of school climate in relation to educational achievement and aspirations of Roma youth by portraying important implications for school success of such an underrepresented minority group across Europe. Promoting positive school climate in the context of interventions will enable schools to achieve positive outcomes among disadvantaged students.
